# Andy Golden: Mentorship through the Years

**DOI:** 10.3390/jdb11040041

**Published:** 2023-11-03

**Authors:** Anna K. Allen, Xiaofei Bai, Edward S. Davis, Amy Fabritius, Aimee Jaramillo-Lambert, Peter A. Kropp, Christopher T. Richie, Jill M. Schumacher, Sanjay Shrestha, Kathryn Stein, Ann K. Corsi

**Affiliations:** 1 Directorate for Biological Sciences, National Science Foundation, Alexandria, VA 22314, USA; anna.allen@howard.edu; 2 Department of Biology, Howard University, Washington, DC 20059, USA; 3 Department of Biology, Genetics Institute, University of Florida, Gainesville, FL 32610, USA; baixiaofei@ufl.edu; 4 LGC Clinical Diagnostics, Gaithersburg, MD 20877, USA; eddavisdna@gmail.com; 5 Department of Molecular and Cellular Biology, University of California, Davis, CA 95616, USA; asfabritius@ucdavis.edu; 6 Department of Biological Sciences, University of Delaware, Newark, DE 19716, USA; anjl@udel.edu; 7 Department of Biology, Kenyon College, Gambier, OH 43022, USA; kropp1@kenyon.edu; 8 National Institute on Drug Abuse, Baltimore, MD 21224, USA; christopher.richie@nih.gov; 9 Department of Genetics, University of Texas MD Anderson Cancer Center, Houston, TX 77030, USA; 10 Medical Sciences, Indiana University School of Medicine-Bloomington, Bloomington, IN 47405, USA; sashrest@indiana.edu; 11 Eunice Kennedy Shriver National Institute of Child Health and Human Development (NICHD), NIH, Bethesda, MD 20817, USA; kathryn.stein@nih.gov; 12 Department of Biology, The Catholic University of America, Washington, DC 20064, USA

The *C. elegans* community mourns the passing of Andy Golden, Ph.D. (1960–2023). At the time of his death, Andy was a Senior Investigator and Section Chief of Genetics of Early Development in the Laboratory of Biochemistry & Genetics in the National Institute of Diabetes and Digestive and Kidney Diseases at the National Institutes of Health. 

**Guest Editor Dedication**

I think it is fairly unusual to dedicate a special issue to one of the authors/editors, but considering I am finishing this special worm project on my own, I believe it is appropriate. Andy passed away unexpectedly before we could complete editing this issue or write the accompanying primer on using worms as a model for development and disease. We were planning together to write a primer that could be used by students and newcomers who are interested in using nematodes for translational research. We were hoping that the review would help readers appreciate the promise nematodes have for understanding human development, disease, and potential therapeutic approaches. I hope you will return to this special issue in the near future to read that forthcoming article. 

I wanted to honor Andy’s memory and his dedication to mentorship (Figure 1), so I invited his former postdoctoral fellows to collaborate with me on this dedication/obituary. You can read their reflections on Andy as an excellent mentor below. The contributions are arranged according to the time period they were in the lab, painting a picture of Andy’s journey as an investigator from the National Cancer Institute (NCI) in Frederick, MD to the National Institute of Diabetes and Digestive and Kidney Diseases (NIDDK) at the National Institutes of Health (NIH) in Bethesda, MD. The fellows themselves range from one of his earliest who just retired, to his most recent fellow who just joined the University of Florida faculty as an assistant professor. These testimonials provide insight into Andy’s work over the years, and, most of all, the experience of being under the tutelage of a marvelous scientist and mentor. 

With only a few Metro stops separating us, my life and Andy’s were intertwined personally and professionally, being friends and colleagues for more than two decades. Like many others, I am left heartbroken at his passing. As a friend told me, “He deserved more time”. I wholeheartedly agree. Although we worked at very different places—he was at a government research institution, and I am at a private religious university—I think we both saw our professions as a vocation to mentor the next generation of scientists. Our mentoring efforts were occasionally intertwined, too. For example, one of my talented undergraduates joined Andy’s lab as a post-baccalaureate fellow and contributed to two publications under Andy’s guidance. Her experience with Andy confirmed her interest in graduate school, and she is now pursuing a Ph.D. at the University of Maryland at Baltimore. In turn, I had the opportunity to mentor several of Andy’s excellent postdoctoral fellows. And, as you will see from their remarks below, Andy’s trainees benefited tremendously from the environment that Andy created both in and out of the lab. 

Andy was also the glue that held our local worm community together. The worm field was founded by individuals who valued cooperation and community, and Andy embodied that spirit in his organization of our Baltimore (Washington) Worm Club. Nearly 30 groups meet every other week year-round to hear two progress reports, usually from trainees, with nearly every principal investigator in attendance. During the pandemic, we only missed a couple of weeks before Andy’s organization and persistence moved us on Zoom and back on track like clockwork. His was the backyard of the social-distancing BBQs. He was the WormBeer organizer (Figure 2) and the creator of our worm quarantine WhatsApp group. Andy made our community special.

In his research, Andy spent many successful years identifying key cell cycle regulators in worms and, in particular, meiotic and early mitotic factors needed for embryonic development. In more recent years, Andy turned his focus to using worms to model rare diseases. He realized that screening for mutations that alleviate the phenotypes of disease-causing mutants engineered in worms would provide insight into possible therapeutic avenues. One of Andy’s interests was in Timothy Syndrome, a multi-system calcium channel disorder that can lead to cardiac arrhythmias. Andy investigated the genetics of Timothy Syndrome, but, amazingly, unlike most bench scientists, Andy got involved at the patient level, too. He became a trusted member of the Timothy Syndrome Foundation and worked tirelessly to understand the natural history behind the disorder in an effort to improve patient quality of life. It was in the rare disease space that Andy and my research became intertwined beyond just our love for *C. elegans.*

In 2014, Andy and I independently received information that would shift the trajectories of both our labs. He learned from the NIH’s Undiagnosed Diseases Program that they had identified several patient mutations in the DNA binding domain of TWIST2, the ortholog of my favorite transcription factor in worms, HLH-8. Even though the mutations were at the identical position in the TWIST2 protein, the different amino acid substitutions caused distinct developmental diseases—Ablepheron Macrostomia and Barber-Say syndromes (AMS and BSS). That same week, I received an email out of the blue from Andrew Wilkie, an expert in craniofacial diseases at Oxford University. He had two patients with mutations in the same DNA binding position as the AMS and BSS patients, but the amino acid substitutions were in TWIST1 instead of the closely-related homolog TWIST2. These patients had more severe phenotypes compared to individuals with other TWIST1 mutations, and the clinicians wanted to know why. My lab had not yet tried CRISPR/Cas9 genome editing. Andy’s lab was busy using the technology for making mutations in worms, but they also needed to develop reagents, such as GFP reporters, for studying them. My group already had such reagents in hand for studying HLH-8. After a long discussion drinking tea under an arbor behind Building 50 at NIH, Andy and I decided to start designing the guide RNAs and work on this together. As a result, we published together my first, and perhaps only, *Human Molecular Genetics* paper, and my lab joined the CRISPR/Cas9 revolution. That work became a “proof-of-principle” for Andy’s future success using *C. elegans* as a model for rare human diseases, and it encouraged Tim Schedl and Stephen Pak (Washington University, St. Louis, MO) to establish the *C. elegans* arm of the Model Organisms Screening Center. Inspiring this successful branch of the Undiagnosed Diseases Network is one of Andy’s most lasting legacies.

Over the years, I have benefited enormously from Andy’s sage advice discussing topics from the intricacies of mapping mutations to managing trainees and everything in between. I wish everyone could have a collaborator and friend like Andy… except now that he is gone, the hole is a little too big, and moving on is a little too impossible. He did, indeed, deserve more time. It is heartbreaking. Rest easy, friend. You are sorely missed. –AKC.

**Reflections on Mentoring from Andy’s Postdoctoral Fellows:**

**Jill M. Schumacher**, Associate Professor of Genetics (retired), University of Texas MD Anderson Cancer Center.

It all started over a beer in 1996. I was a postdoctoral fellow in Peter Donovan’s lab at the NCI in Frederick, MD. My plan was to use mammalian cells and mouse models to study a novel kinase that was localized to centrosomes during mitosis. I had just arrived in Frederick and was getting used to a new environment and lab. I attended a party at a fellow scientist’s home and met Andy Golden (I think for the first time), the worm guy down the hall from Peter’s lab. Given his friendly and approachable nature, we quickly fell into a conversation. Andy told me that the worm genome project had revealed that the kinase I was studying (now called Aurora A) was strikingly homologous to two genes in *C. elegans*. And excitingly, there was a brand new and very efficient technique to deplete gene products in *C. elegans* embryos simply by injecting double-stranded RNA corresponding to the gene of interest. Intrigued by the possibilities and encouraged by Andy’s warm and open personality, I agreed to try my hand at a few experiments in *C. elegans*. The rest is history. This conversation started a decades long friendship and collaboration with Andy that I will be forever grateful for. 

I quickly realized that *C. elegans* had everything I was looking for: an early embryo with beautiful spindles, facile forward and reverse genetics, a host of transgenic strains for live imaging, and a welcoming scientific community. I started spending most of my time in Andy’s lab and switched to his lab officially when Peter Donovan moved his lab to Philadelphia. I was lucky enough to join Andy’s lab just as he was hitting his stride as a junior PI. In addition to the science, my greatest memory of Andy’s lab was the laughter. Andy was hilarious. Jokes were constantly flying around the lab, many about my propensity to work too fast and drop piles of worm plates, but it was always in good humor and with kind-heartedness. I learned so much from Andy and his humanness. He was always kind, no matter what was happening in his own life and was always concerned about the well-being of those around him. He was patient, spending hours teaching me how to inject worms, and not getting too exasperated when we would have to do it all over again the next day when nothing survived the trauma I inflicted. Andy was generous. My “side” project in Andy’s lab quickly led to two publications and a rich field for further study of the Aurora kinases (AIR-1 and AIR-2 in *C. elegans*). Andy and I both hit the job market at the same time in 1998, and he assured me that without question the Aurora project was mine to take with me to my new lab. 

Andy and I each found homes at our new institutions in 1999. Andy at NIDDK, and I at the MD Anderson Cancer Center. We continued to collaborate for years and remained close friends. It was always a delight to see Andy at worm meetings. Many more beers and more great times. Personally, Andy attended my wedding and both my son and my daughter’s Bar and Bat Mitzvahs in Pittsburgh (Figure 3). I was always welcomed to his and Alex’s home in Ellicott City with open arms whenever I had an occasion to visit. Over the years, one story sticks with me. My first pregnancy ended in a 2nd trimester loss. After this loss, Andy called me daily to check in with me. It was so comforting to hear from someone during such a difficult time. Andy was such a mensch.

To this day, I am amazed that a beer at a party had such a profound impact on my life and career. My heart goes out to Alex and Zoe and Andy’s brother Hal. Andy was golden through and through. I miss him.

**Edward S. Davis**, Field Application Scientist, LGC Clinical Diagnostics.

For me, Andy was first a friend, then a mentor, but always a friend! I met Andy during my first postdoc in NCI-Frederick as he was starting up his own lab there, and because we both lived near each other, we would occasionally carpool. Our car discussions were often scientific in nature and very stimulating—I was working on meiotic recombination in yeast but I was really fascinated with his developmental biology studies in worms—but they also delved into the personal, such as what was the latest food my newborn son was eating and how Andy’s beloved dog Abbie was doing.

Later I joined Andy’s lab at NIDDK and continued my interests in meiosis, but this time in *C. elegans*. That period was the most gratifying of my career. The work was exciting, the model was cool, the NIH campus, on which I was doing my second tour of duty, was stimulating and beautiful, and, most importantly, Andy was a first-class mentor, fostering a productive and positive research environment with a calm, steady hand. I will always cherish my experience in Andy’s lab.

**Christopher T. Richie**, Staff Scientist, Manager of the Genetic Engineering and Viral Vector Core, National Institute on Drug Abuse.

I will remember Andy for his generosity and patience, as well as his work ethic. When I joined his lab in 2003, it was just a single room in Building 8 which contained all the office and bench space. His sense of humor and community set a good example for co-existing in such tight quarters. He was always accessible and willing to talk about anything, even if investing in that conversation meant that he would be staying late to get that final item on his to-do list done. I am grateful for the time he spent on me as I continued my larval development as a scientist. 

**Katie Stein**, Program Director, *Eunice Kennedy Shriver* National Institute of Child Health and Human Development (NICHD).

[Comments and views of the author(s) do not necessarily represent the views of the Eunice Kennedy Shriver NICHD.

Andy’s kindness and genuine interest in connecting with people were the qualities that set him apart as a mentor and formed the foundation of his impact. Andy was great at questions. In part, this was because he generously assumed the best about people—he was not going to judge anyone for a ‘stupid’ question, which made it easy to approach him for guidance on a challenging concept or the design of an experiment. He also never seemed to have anything to prove, so his questions were critical and clear, and were never posed to make someone look bad or, conversely, to make himself look smart.

Andy’s mentorship was crystallized in his leadership style, which I would call ‘leading by harmony’. This harmony had the effect of bringing people together and instilling the feeling that we had a shared goal. I used to think that I was just incredibly lucky to have been in Andy’s lab at that particular moment, with just the right combination of people that made going to work inspiring and fun. Now, though, I think that magic probably came from Andy himself and his practice of harmony—he made the default program a spirit of acceptance, camaraderie, mischief, and always (always!) plenty of snacks, which was just a physical manifestation of the abundant, welcoming tone that he projected. I am grateful to have known him and to have had him as a keystone person in my life.

**Anna Allen**, Program Director, Directorate for Biological Sciences, National Science Foundation; Associate Professor Emeritus, Howard University.

Andy Golden was my postdoc mentor and my friend. For the past 15 years, he has been my #1 scientific champion. He was the type of scientist we should all strive to be. Smart, kind, generous, funny (Figure 4). The worm community, the world, and I are all better because he was here. I will truly, truly miss him. I want to take my space here to share some short vignettes about Andy so that others can appreciate the type of person and mentor he was.

**All in**—Andy was the type of mentor who went all in. He gave as much of himself to you as you wanted to take. You were not just an individual working in his lab, you were offered (should you want it!) the opportunity to be his colleague, peer, and friend. Importantly, his mentorship and friendship did not just last while you were physically working within his laboratory. Long after I left his lab, I would get random emails and text messages from Andy checking up on me. I knew I could always call him if I wanted to discuss something that was puzzling me (either experiment related, mentoring related, or personal related). Even though it has been over 2 months, I still cannot believe he is no longer just a phone call away.

**Supportive**—Andy not only encouraged and guided his mentees in science, but also in life and life decisions. He was always willing to provide technical advice and go over exciting (or troubling!) scientific results. He especially loved complex genetic crosses! But what I want to highlight, which can be unusual in the scientific community, is how supportive Andy was of your life outside of the laboratory. In 2009, I remember being nervous going into his office to tell him I was pregnant with my first child. His reaction was a huge smile, a big hug, and a “being a parent is the best and most worthwhile job”. This was just the response I needed as a female scientist who desired to be both a successful scientist and an involved mother, and was terrified as to whether I could realistically be both. Andy and I had many discussions about parenting both after the birth of my first child and then my second child while I was a member of his laboratory. One thing I remember keenly is Andy’s devotion to his own family, in particular his daughter. Andy prioritized family time; he was the dad who attended all the swim meets. He really modeled how one could be both a successful scientist and an involved parent.

**Humanity**—Andy showed humanity to all. He always looked for the good in people and focused on their potential. He treated everyone as equals, from the summer undergraduate intern to the 4th year postdoctoral fellow. He respected those who worked in his laboratory and recognized their successes and accomplishments. He did not focus on or emphasize an individual’s mistakes. I made many mistakes during my time in Andy’s lab, the first being just weeks after joining. (If you are wondering…. one needs to use RNAi plates not regular MYOB plates when doing an RNAi experiment!). Andy dealt with my first mistake in the lab by simply informing me that life is full of mistakes, and one just needs to learn from their mistakes and move on.

Andy was an amazing person. I am eternally grateful to have joined his lab in 2008. He has believed in me, guided me, and mentored me since then. Andy was always willing to help anyone, and this extended to those members of my own laboratory at Howard University. One of the final conversations I had with Andy was regarding my decision to move from academia to the National Science Foundation. He was only encouraging regarding this career change, reiterating how he knew there was not only one way to help advance science and that it seemed like all my past experiences were leading me to NSF. The call ended with “I’m proud of you”.

Andy—since I never got a chance to tell you then, I am proud of you and your legacy. Thank you for being an amazing mentor and friend. #BeLikeAndy

**Amy Fabritius**, Project Scientist, University of California-Davis.

When I think of Andy, I think of him celebrating. Whether it was a successful experiment or a birthday, Andy encouraged people to celebrate. His lab and surrounding labs came together frequently for lunches or parties (especially with good food). I appreciate the sense of community and positive environment he created, and I hope I can pass some of that on.

**Aimee Jaramillo-Lambert**, Assistant Professor, University of Delaware.

It is hard to put into words the many qualities and things that Andy did that made him a great mentor. He was kind. He was supportive. And he was funny! Andy was open to people from all backgrounds and genuinely interested in each person as an individual. I was always amazed about how much he remembered about my family life; although he did like to joke that he wanted me to draw out my complicated family tree so that he could keep track. He understood that his mentees are whole people with both scientific and personal interests and was eager to share in both our scientific triumphs and personal accomplishments. This attribute also came out in how supportive he was in our scientific and personal goals. I had my first child while doing my postdoctoral work in his lab and Andy hosted my baby shower. For professional development, Andy supported my goal of entering academia and was willing to let me take some time away from the lab to gain teaching experience both within the intramural NIH system and externally at a local university. His support extended well into my independent career. Over the past six years, he continued to give advice on how to run a lab, scientific feedback, and he even served as the outside committee member for a Ph.D. student in my lab. I will end with how fun Andy made working in the lab every day. From the well-stocked snack shelf, his silly jokes, games (e.g., Loser’s Lunch), to his “Still got it” every time he did a successful PCR, working with Andy was a pleasure. It still hits me hard when I think of some scientific question that he would know about or if something comes up that I would have liked to share with him. I miss him deeply and hope to emulate elements of Andy as I mentor trainees. —AJL

**Peter Kropp**, Assistant Professor, Kenyon College.

As I reflect on my time as a postdoc with Andy, I struggle to appropriately commemorate him both as a mentor and a friend. Andy was such a rich person, and personality, that he impacted all of those around him in meaningful ways. He will always be remembered for his kindness and generosity, for his love of pranks and palindromes, and for his tireless dedication to making work fun. Of course, he was also a great scientist. I left his lab to start my own in 2022. Despite the fact that we had ongoing collaborations, our continued conversations typically skewed towards personal matters. Andy cared deeply about everyone as individuals, not just their professional roles or relationship in a mentor-mentee structure. I and many others consider Andy a close friend, not just a mentor or colleague. Above all, I think that is his lasting impact.

Andy took a chance on me. I had never picked a worm prior to joining his lab, and we came from very different scientific backgrounds. What I did not know at the time of joining Andy’s lab was that my *C. elegans* naiveté and our different approaches to answering the same question would result in creative experimental design and many fruitful projects. He taught me classical genetics, about thoughtful experimental design, and how to be the best collaborator possible, all of which now shape how I approach experiments, teach, and mentor. I am a better scientist, and person, for having worked with him for nearly five years.

As great as Andy was as a mentor, the memories of him that jump to mind have nothing to do with science. I am still struck by how humble, unpretentious, and sometimes just silly he was. He brought “embarrassing dad” energy to everything that he did, whether it was sporting his infamous Crocs, wearing a multicolored jester hat all day, or oversharing just a bit. His disarming energy made folks more comfortable and helped to break down normal barriers in academia. Perhaps one example is that during my interview to join his lab, he took me to a lesser-known cafeteria on the NIH campus that can generously be described as a greasy spoon. Many folks avoid this cafeteria, but Andy loved the tuna melt. In his mind, that was all that was needed to impress me—or maybe he just wanted the tuna melt that day. Regardless, it set the tone for the rest of our time together: good conversations while enjoying the simple things.

Andy’s professional legacy is the people that he trained, collaborated with, and inspired. He will be missed by many and the worm community has suffered a great loss. But, no one who knew him will ever be able to look at a pair of Crocs, a Philadelphia Eagles hoodie, or a Technicolor shirt without thinking of him. —PAK

**Sanjay Shrestha**, Research Staff, Indiana School of Medicine-Bloomington.

The passing away of Andy was shocking to me. It was too early for him. 

Andy gave me an opportunity to learn and widen my scientific horizons. The time I spent in Andy’s lab was transformative and it is where I got a chance to learn how *C. elegans* has been a powerful tool in understanding fundamental biological processes. Andy’s idea of using suppressor screen strategy in worms to explore therapeutic potentials to address human rare genetic diseases, and using CRISPR/Cas9 genome editing tools to explore rare monogenic diseases really fascinated me. Working on those projects was exciting for me as they are stepping stones to bridge basic to bedside research. In my postdoctoral job interview he told me, ‘Create your own scientific niche’, which I still carry in my heart.

Coming from a different scientific background, Andy mentored and guided me through worm genetics with great patience. He even lent me his own book to understand worm genetics. We used to attend Worm club every two weeks in Baltimore. The experience was amazing. I still remember that crowd of intelligent minds. The training I got from Andy’s lab added another important layer of expertise which allowed me to do genetic manipulations in human cells. In addition to being a remarkably dedicated and committed scientist and a mentor, what I admired about him is that he was a very generous, very kind-hearted, honest, and down-to-earth person. I am thankful to Andy for all his support and guidance. Now, Andy is no longer with us, but memories remain. His passing away is a great loss and created a deep void for the scientific community. Anybody who knows Andy will greatly miss him. 

**Xiaofei Bai**, Assistant Professor, University of Florida.

With a heavy heart, I tried to put into words my memories with Andy and the time we spent together at NIH (Figure 5). I am filled with both sadness and gratitude as I reflect on being Andy’s last postdoctoral fellow after his remarkable twenty-three-year career at NIDDK/NIH. My first encounter with Andy was in 2016 when I was a Ph.D. student at the University of Tennessee, Knoxville. Attending his seminar on modeling human rare diseases in the nematode model was a turning point for me. Andy’s talk not only fascinated me with science but also reminded me of the unfortunate reality back home in China. I grew up in a conservative community in the remote northwestern part of the country, where a high frequency of infants with congenital birth defects or disabilities were born due to consanguineous marriages. Witnessing their struggles without effective treatments left a profound impact on me. Andy’s work and his presentation ignited a strong desire in me to study disease-associated genes and search for potential therapies. I knew then that working with Andy as a postdoctoral fellow was my dream. In 2017, that dream came true when I had the opportunity to give a platform presentation at the *C. elegans* international meeting. Andy was impressed by my presentation and offered me a postdoc position once I completed my graduate studies. Since then, we cultivated a close and productive communication, envisioning the potential of using *C. elegans* as a powerful tool to model human rare diseases.

The summer of 2018 marked the beginning of my journey as a postdoctoral fellow in Andy’s lab. Under his mentorship, I received comprehensive training in research, career development, scientific writing, mentorship, and lab management. Andy’s nurturing nature created a warm and friendly lab environment where I felt secure, happy, and confident to explore intriguing scientific questions. He fostered my growth as an independent scientist and future principal investigator. Throughout my postdoctoral training, Andy encouraged me to pursue various projects focused on disease-associated genes, including those related to the mechanotransductive ion channel PEZO-1, the lipid droplet protein Seipin, and the fatty acid synthase FASN-1, among others. These projects not only expanded my scientific horizons but also deepened my appreciation for *C. elegans* as a genetic model and experimental platform to explore the molecular and cellular mechanisms of disease-associated genes.

Andy’s mentorship extended beyond the lab, as he connected me with other worm researchers, including my co-mentors Dr. Erin Cram at Northeastern University and Dr. Harold E. Smith at NIDDK, as well as many other experts. Despite his busy schedule and long commute, Andy always found time to support me and provide valuable feedback on my work. He not only guided me as a scientist but also helped me navigate cultural differences and professional growth. After we published our first two papers, Andy encouraged me to take on the role of a mentor to guide and train younger trainees in the lab, including a few summer interns and post baccalaureate fellows. These experiences allowed me to develop into a confident and independent scientist, revealing my potential to become a principal investigator. 

When the pandemic struck in 2020, Andy showed genuine concern for my family in China, and he frequently checked in on their well-being and offered his support. His humble and caring nature endeared him to everyone around him. Despite the challenges of the pandemic, Andy made sure to maintain regular Zoom meetings, phone calls, and group texts with the lab, ensuring everyone’s well-being and keeping us engaged, even while facing his own medical concerns. 

One of the most defining moments of Andy’s mentorship was when I applied for my first NIH grant, the K99/R00. He dedicated an enormous amount of time to support my application, by writing a compelling supporting letter and helping me design my career development and research plans. The day I received the grant, his pride and excitement were evident, and we celebrated with a heartfelt high-five. I can still vividly recall his joyful expression and warm smile, which brought tears of happiness to my eyes. During the early stage of my fourth-year postdoc training, Andy and I had a significant one-on-one meeting, which we held bi-weekly. He expressed his admiration for the progress I had made and my growth in recent years, and he strongly encouraged me to start preparing to apply for a faculty position. We both agreed that this career path would be the best fit for me. During the meeting, Andy provided valuable insights into the job-hunting process and stressed the importance of key application materials. With a warm smile, he said, “You will do great, Bai. I have no doubt you will end up at a good place to start your own lab”. With Andy’s unwavering support and glowing recommendations, I eventually signed an offer with the University of Florida and officially began my own lab in August, 2023.

Before my move to Florida, Andy and I had a two-hour one-on-one meeting to go over what I needed to do and prepare as a new principal investigator. He generously offered anything I might need for my new lab. On the day before he was taken to the hospital, I visited the lab to discuss a manuscript we were planning to submit. Andy seemed a bit melancholic and thanked me for everything I had contributed to the lab. I playfully responded, saying I would be back for him whenever he needed me. Little did I know that would be the last time I saw him. Andy’s passing came unexpectedly and left an immense void in my heart. He was incredibly generous and kind, and I cannot express enough gratitude for everything he did to escort me to where I am today. He left us too soon! When I set up my own lab, I took pictures, wishing I could share them with Andy and see his prideful smile. It brings tears of sadness to my eyes. Rest in peace, Andy; you are deeply missed. —Bai

## Figures and Tables

**Figure 1:**
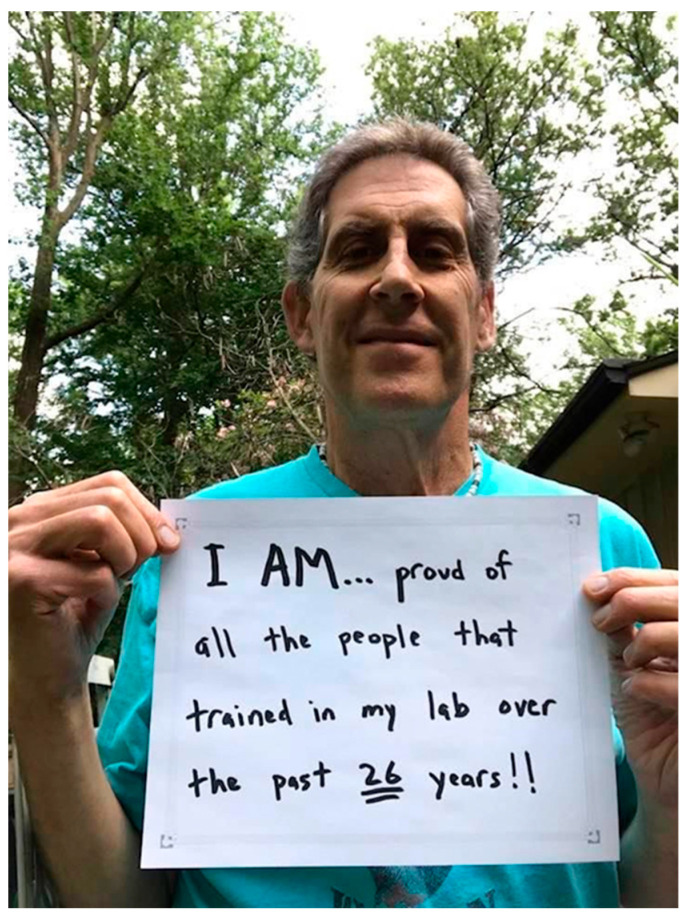
Andy Golden—in typical fashion celebrating himself as a mentor. Photo credit: Yashira Ortega-Sustache.

**Figure 2:**
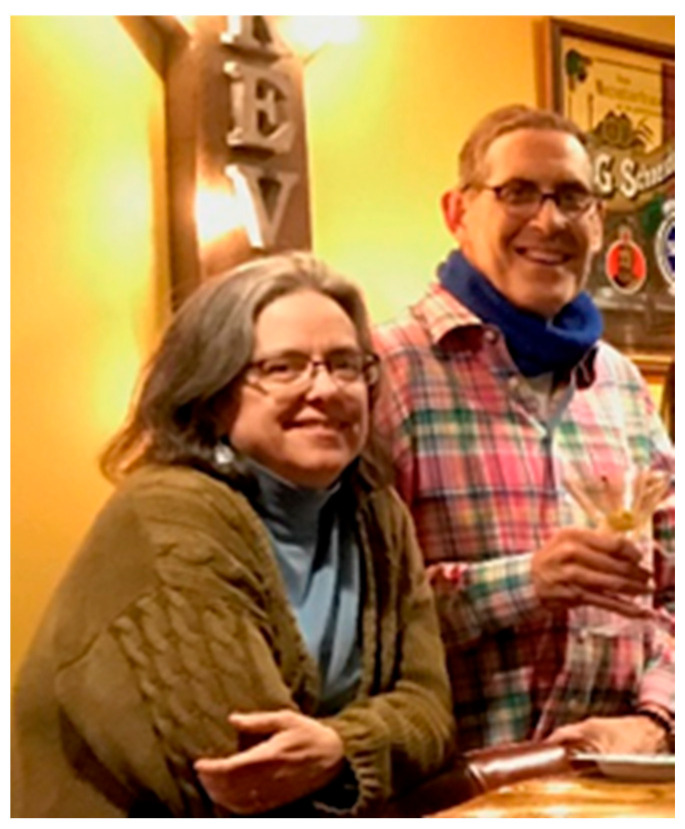
Ann and Andy at a “Worm Beer”. Photo credit: Laurie O’Connell.

**Figure 3:**
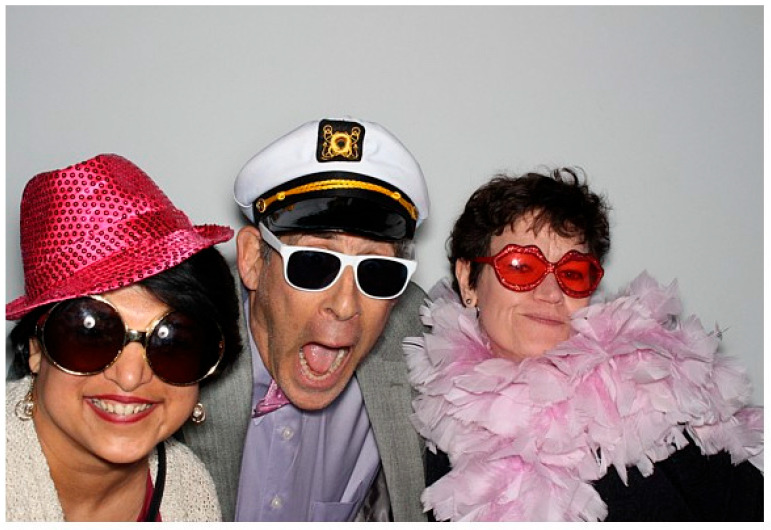
Silly side of Andy at the Bat Mitzvah of Jill’s daughter Emily Gutstein in January 2018. (L–R) Swathi Arur, Andy, and Jill. Photo Credit: Jill Schumacher.

**Figure 4:**
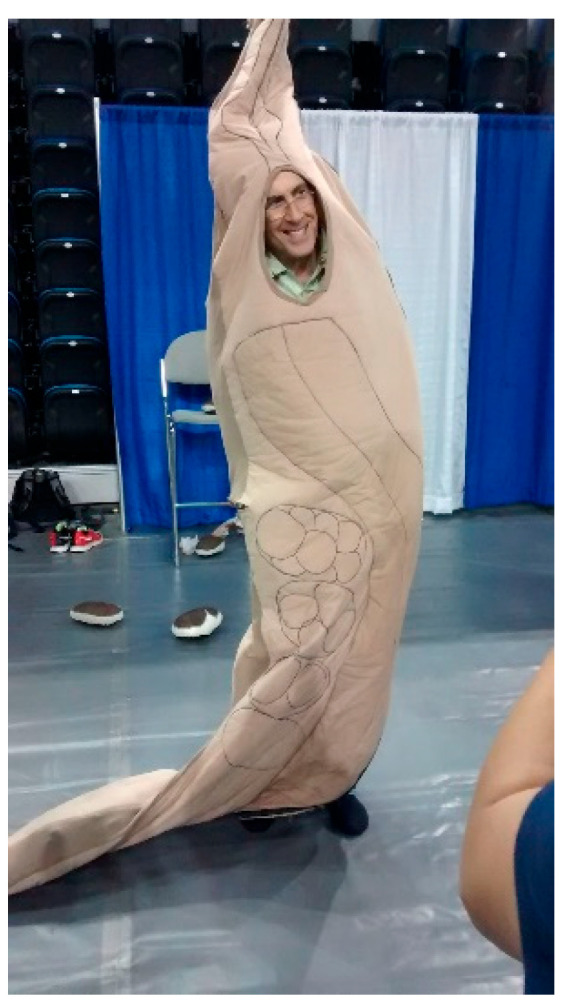
Andy donning the worm costume that was part of the worm art show at the International *C. elegans* meeting in June 2015. Photo credit: Anna Allen.

**Figure 5:**
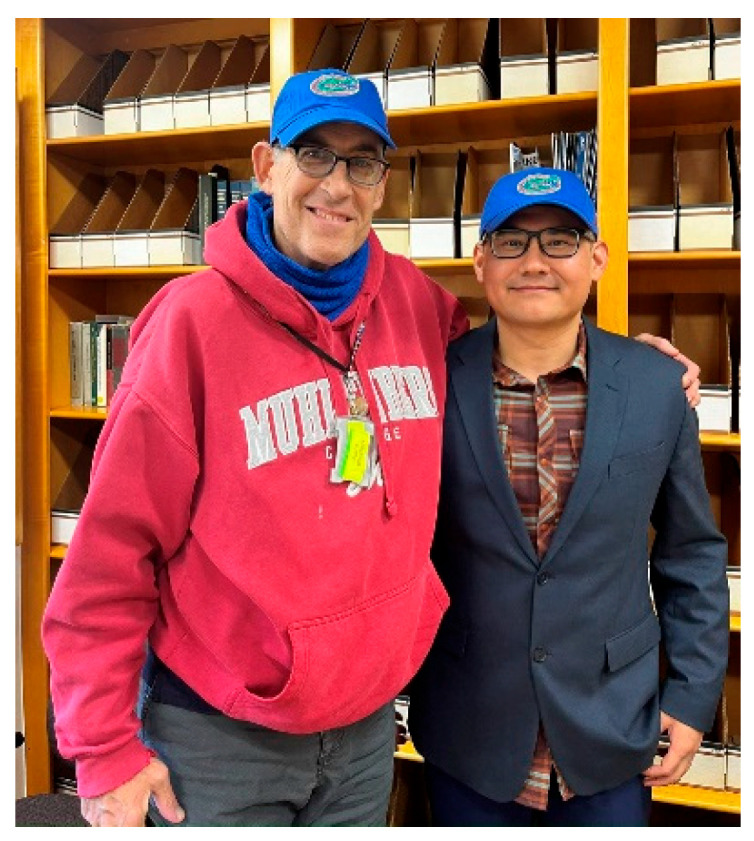
Andy and Bai. Photo Credit: Sydney Kelly.

